# The value of FATS expression in predicting sensitivity to radiotherapy in breast cancer

**DOI:** 10.18632/oncotarget.16630

**Published:** 2017-03-28

**Authors:** Jun Zhang, Nan Wu, Tiemei Zhang, Tao Sun, Yi Su, Jing Zhao, Kun Mu, Zhao Jin, Ming Gao, Juntian Liu, Lin Gu

**Affiliations:** ^1^ Department of Breast Surgery, Tianjin Medical University Cancer Institute and Hospital, National Clinical Research Center for Cancer, Tianjin's Clinical Research Center for Cancer, Key Laboratory of Cancer Prevention and Therapy, Tianjin 300060, China; ^2^ Department of Endoscopy, Tianjin Medical University Cancer Institute and Hospital, National Clinical Research Center for Cancer, Tianjin's Clinical Research Center for Cancer, Key Laboratory of Cancer Prevention and Therapy, Tianjin 300060, China; ^3^ Department of Breast Surgery, Hebei Province Cangzhou City Nanpi People's Hospital, Cangzhou 061500, China; ^4^ Department of Thyroid and Neck Tumor, Tianjin Medical University Cancer Institute and Hospital, National Clinical Research Center for Cancer, Tianjin's Clinical Research Center for Cancer, Key Laboratory of Cancer Prevention and Therapy, Tianjin 300060, China

**Keywords:** breast cancer, radiotherapy, FATS, biomarker

## Abstract

**Purpose:**

The fragile-site associated tumor suppressor (FATS) is a newly identified tumor suppressor involved in radiation-induced tumorigenesis. The purpose of this study was to characterize FATS expression in breast cancers about radiotherapy benefit, patient characteristics, and prognosis.

**Results:**

The expression of FATS mRNA was silent or downregulated in 95.2% of breast cancer samples compared with paired normal controls (*P* < .0001). Negative status of FATS was correlated with higher nuclear grade (*P* = .01) and shorter disease-free survival (DFS) of breast cancer (*P* = .036). In a multivariate analysis, FATS expression showed favorable prognostic value for DFS (odds ratio, 0.532; 95% confidence interval, 0.299 to 0.947; (*P* = .032). Furthermore, improved survival time was seen in FATS-positive patients receiving radiotherapy (*P* = .006). The results of multivariate analysis revealed independent prognostic value of FATS expression in predicting longer DFS (odds ratio, 0.377; 95% confidence interval, 0.176 to 0.809; *P* = 0.012) for patients receiving adjuvant radiotherapy. In support of this, reduction of FATS expression in breast cancer cell lines, FATS positive group significantly sensitized than Knock-down of FATS group.

**Materials and Methods:**

Tissue samples from 156 breast cancer patients and 42 controls in tumor bank were studied. FATS gene expression was evaluated using quantitative reverse transcription polymerase chain reaction (qRT-PCR). FATS function was examined in breast cancer cell lines using siRNA knock-downs and colony forming assays after irradiation.

**Conclusions:**

FATS status is a biomarker in breast cancer to identify individuals likely to benefit from radiotherapy.

## INTRODUCTION

Breast cancer is a leading cause of cancer death among women, second only to lung cancer. Although mammography has improved early detection and increased the 5-year survival rate to 98% for breast cancer, the survival rate drops dramatically to 83% for patients initially diagnosed with regional spread and to 26% for those with distant metastases [1]. Radiotherapy continues to play a key role in the management of breast cancer. Despite this, radioresistance manifest by locoregional failure after radiotherapy remains a challenge [2]. In addition, increased risk for second malignant neoplasms (SMNs) after relative lose-dose radiation is raising more and more concern [3–6]. Given that there is no threshold dose below which no increase in risk to health is posed [5]. The rationale design of therapeutic strategies is needed. However, little is known about reliable molecular tumor markers to identify individuals likely to benefit from radiotherapy.

The hallmark of cancer is genomic instability, resulting from defects in cell-cycle checkpoints and DNA repair. Through genome-wide approach, we have identified a tumor suppressor, the fragile-site associated tumor suppressor (FATS), at a genomic region susceptible to DNA damage induced by radiation or replication stress. FATS gene plays a critical role in monitoring cell-cycle checkpoints and thereby maintaining genome integrity [7]. To evaluate the relevance of FATS to breast cancer, and to explore the clinical significance of FATS in prognosis of breast cancer, we conducted a study to assess the level of FATS expression quantitatively in breast cancer.

## RESULTS

### Expression of FATS mRNA transcripts in breast cancer samples

**Figure 1:**
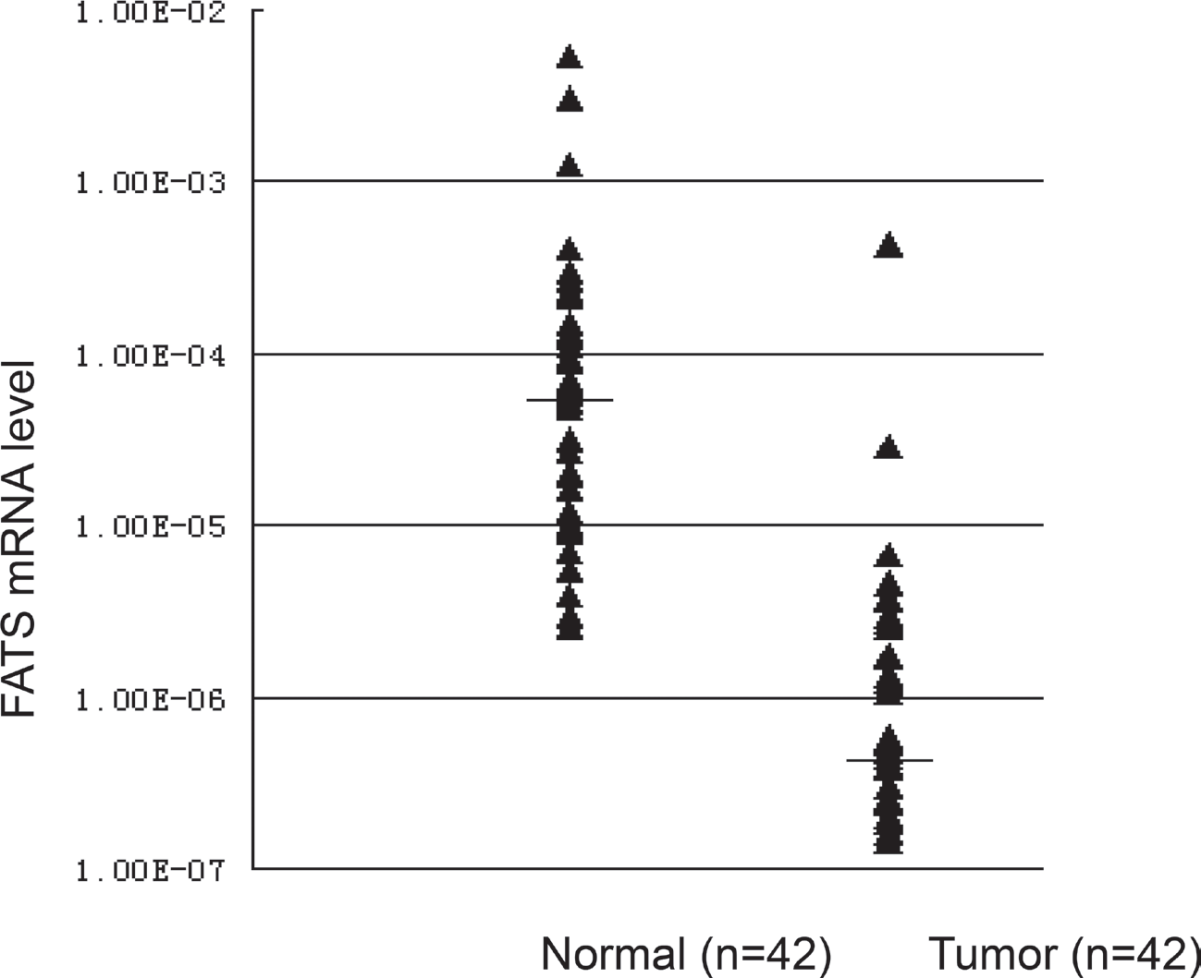
The expression levels of FATS mRNA in breast cancers and paired normal breast tissues FATS expression was silent or downregulated in 95.2% (*n* = 42) breast cancer specimens. The median level of FATS mRNA in normal breast tissues and breast cancers was 5.56E-05 and 5.01E-07, respectively.

Previous data have shown that FATS is a tumor suppressor at the genomic region susceptible to DNA damage induced by radiation or replication stress [8]. Because FATS is expressed in mammary gland but extensively silent or downregulated in mouse tumors [7], it can be expected that its expression might also be altered in human breast cancer. To test this hypothesis, we analyzed FATS expression in paired normal and tumor tissues using quantitative reverse transcription polymerase chain reaction (qRT-PCR). Among the 42 paired breast cancers in the present analysis, 95.2% were significantly downregulated or silent (Figure 1). After normalization with a housekeeping gene expression, these alterations in FATS expression in breast tumor tissues were confirmed. In contrast to the median level of FATS mRNA (5.56E-05) in normal breast tissues, the average level of FATS expression (5.01E-07) in paired breast tumors was 100-fold decreased (Figure 1), strongly indicating the low expression of FATS in human cancer.

### Clinical association of FATS expression

To evaluate the significance of FATS expression in breast cancer, tumor samples from 156 cases were obtained from Tumor Bank at Tianjin for detecting FATS expression by qRT-PCR. Notably, the FATS mRNA levels in 155 out of 156 (99.4%) breast tumor samples were lower than the average level of FATS expression in normal breast tissue (Figure 1 and data not shown), and downregulation of FATS expression in breast cancer was statistically significant (*P* < 0.001, Table 1).

**Table 1:** Associations of FATS expression with clinical characteristics

Characteristic	Cases	FATS expression		*P*
		negative	positive	
Breast tissue				
Normal	42	2	40	0.000*
Cancer	156	73	83	
Age (years)				
< 50	66	32	34	0.662
≥ 50	90	41	49	
Menopausal status				
Pre/peri-	87	39	48	0.585
Post-	67	33	34	
Missing	2	1	1	
Tumor size (cm)				
≤ 2 cm	20	6	14	0.290
2–5	106	53	53	
>5 cm	29	13	16	
Missing	1	1	0	
Clinical stage				
I	12	3	9	0.233
II	104	53	51	
III	36	17	19	
Missing	4	0	4	
Lymph node status				
−	63	30	33	0.865
+	93	43	50	
Lymph node positive number				
0	63	30	33	0.714
1–3	38	15	23	
4–9	26	12	13	
≥ 10	30	16	14	
Nuclear grade				
I	2	0	2	0.01*
II	114	48	66	
III	36	24	12	
Missing	4	1	3	
ER				
Negative	63	30	33	0.774
Positive	84	38	46	
Missing	9	5	4	
PR				
Negative	85	40	45	0.820
Positive	62	28	34	
Missing	9	5	4	
Her2				
Negative	100	41	59	0.062
Positive	47	27	20	
Missing	9	5	4	

*Difference was statistically significant.

ROC curves were constructed by plotting sensitivity versus specificity to determine the cut-off value (5.3E–07) for distinguishing FATS status (negative/ positive) [9–11]. Among 156 cases, 83 cases were grouped as FATS-positive and 73 cases were regarded as FATS-negative, according to the cut-off value. The clinical and pathologic characteristics and the FATS status in breast cancer patients were shown in Table 1. A lower level of FATS expression in breast cancer was associated with a higher nuclear grade (*P* = .01), and there were no correlations between FATS mRNA levels and other clinicopathologic factors including clinical stage, tumor size, lymph node status, ER status and PR status. There was a trend of inverse correlation between FATS expression and Her2 status, although these differences were not significant (*P* = .062).

### FATS mRNA level is an independent prognostic biomarker for favorable clinical outcome

**Figure 2:**
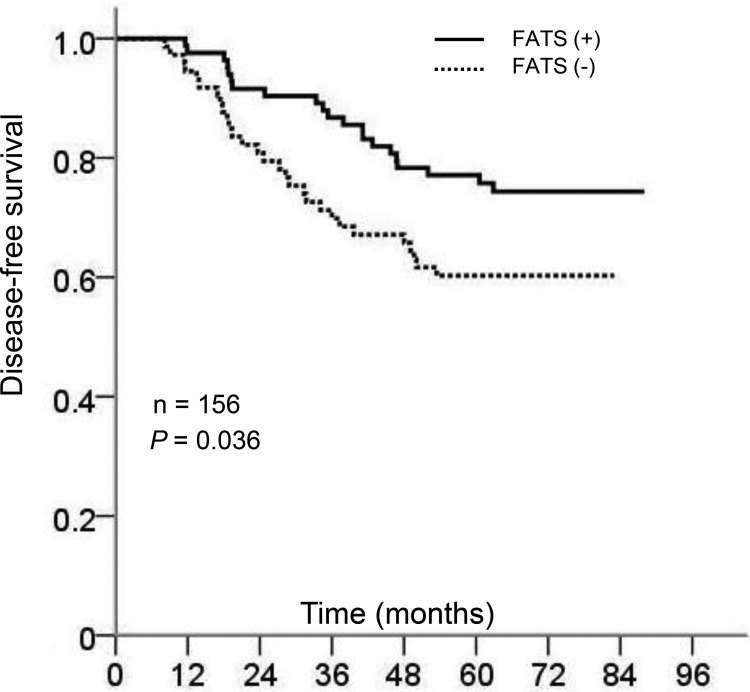
Kaplan-Meier curves of survival probability for breast cancer patients with or without FATS expression *P* = .036 (*n* = 156).

All corresponding cases (*n* = 156) were followed up for more than 5 years until death or the end of the study. In Kaplan-Meier log rank analysis, the individuals with FATS-negative breast cancer had a significant shorter disease-free survival (DFS) time (*P* = .036), as showed in Figure 2 and the univariate analysis in Table 2. To note, although the difference was not statistically significant, the trend that 5 years overall survival (OS) of FATs-positive breast cancer patients was higher than FATs-negative patients could be observed (data not shown). As usually observed, the presence of ER was favorable prognostic factors (*P* = .007). In contrast, the high clinical stage and positive status of axillary lymph node were unfavorable prognostic factors (*P* = .009 and *P* = .005, respectively).

**Table 2:** Univariate and multivariate analysis with prognostic factors in breast cancer for disease-free survival

Category	Variables	Univariate analysis	Multivariate analysis		
		*P*	OR	95% CI	*P*
All cases(*n*= 156)	Clinical stage	0.009*	1.706	0.946 to 3.078	0.076
	Lymph node status	0.005*	2.182	1.007 to 4.727	0.048*
	ER	0.007*	0.473	0.255 to 0.878	0.018*
	PR	0.043*	0.757	0.390 to 1.468	0.410
	Radiotherapy	0.071	1.158	0.586 to 2.290	0.673
	FATS status	0.036 *	0.532	0.299 to 0.947	0.032*
					0.032*
SubgroupreceivingRadiotherapy(*n*= 81)	Clinical stage	0.020*	2.010	0.980 to 4.120	0.057
	ER	0.000*	0.291	0.136 to 0.649	0.002*
	PR	0.091	0.646	0.272 to 1.533	0.322
	FATS status	0.006*	0.377	0.176 to 0.809	0.012*

DFS disease-free survival, OR odds ratio, CI confidence interval, ER estrogen receptor, PR progesterone receptor.

*Difference was statistically significant.

We further investigated whether FATS mRNA levels were an independent prognostic factor using Cox regression. In multivariate analysis (Table 2), as expected, lymph node positive and high pathological stage were associated with poor DFS (OR, 2.182; 95% CI, 1.007 to 4.727; *P* = .048, and OR, 1.706; 95% CI, 0.946 to 3.078; *P* = .076, respectively), whereas positive ER status was significantly associated with favorable clinical outcomes (OR, 0.473; 95% CI, 0.255 to 0.878; *P* = .018). Similarly, FATS status was independently significantly associated with prognosis for DFS (OR, 0.532; 95% CI, 0.299 to 0.947; *P* = .032). Unexpectedly, PR status was not an independent prognostic factor in multivariate analysis (*P* = .410, Table 2), reflecting the challenges to redefine a role for PR in breast cancer [12–14].

### FATS expression and sensitivity to radiotherapy

**Figure 3:**
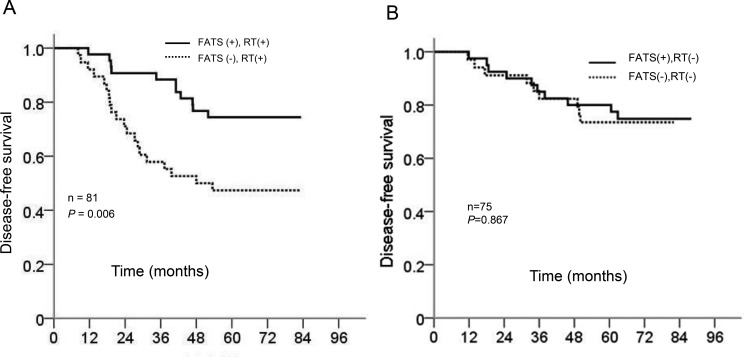
(**A**) Kaplan-Meier curves of survival probability for patients with the treatment of adjuvant radiotherapy (RT) in relation to FATS status. *P* = .006 (*n* = 81). (**B**) Kaplan-Meier curves of survival probability for patients without the treatment of adjuvant radiotherapy (RT) in relation to FATS status. *P* = .867 (*n* = 75).

Given that FATS is involved in DNA damage response and plays a critical role in maintaining genomic stability under DNA damage [7], we next examined whether FATS mRNA level could be a predictive marker for radiotherapy in our cohort of breast cancer patients. Eighty-one (51.9%) of 156 patients with breast cancer were subjected to adjuvant radiotherapy after surgery. Radiotherapy significantly increased DFS of breast cancer patients with positive FATS status (*P* = .006) in comparison to that of FATS-negative patients (Figure 3A). Multivariate logistic regression analysis revealed that FATS status was a more valuable predictor of DFS (OR, 0.377; 95% CI, 0.176 to 0.809; *P* = .012) for breast cancer patients receiving radiotherapy (Table 2). Because radiotherapy by itself was not an independent prognostic factor (Table 2), these results indicated that FATS expression possessed independent prognostic values in predicting sensitivity to radiation treatment of breast cancer.

**Figure 4:**
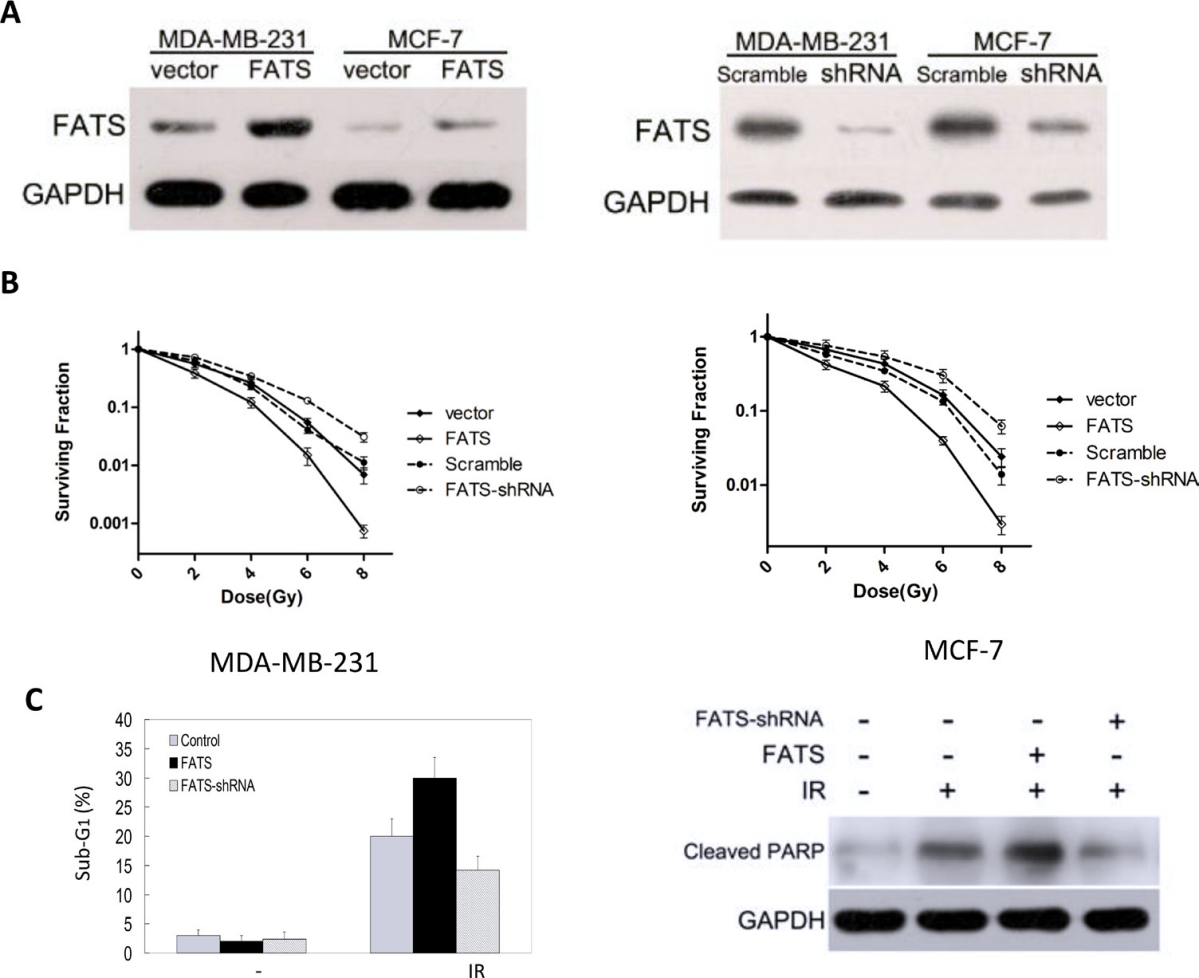
FATS mRNA level in breast cancer cells predicts the sensitivity to radiation treatment (**A**) FATS was effectively silenced using siRNA, as demonstrated using Western blotting of MCF7 and MDA-MB-231 transfected lysates. (**B**) FATS positive group significantly sensitized than Knock-down of FATS group in both cell lines, as demonstrated using colony forming assay. (**C**) MDA-MB-231 cells were transfected with a FATS-expressing vector, siRNAs targeting FATS or an empty vector (control), respectively. After transfection for 24 h, cells were subjected to 6 Gy ionizing radiation (IR) treatment, and flow cytometry analysis was performed 72 h later. The percentage of sub-G1 phase from three individual experiments was plotted for comparison. The data were represented as means ± s.d. The expression of FATS mRNA was detected by reverse transcription polymerase chain reaction. MDA-MB-231 cells were transfected with or without FATS. After transfection for 24 h, cells were subjected to IR (6 Gy) treatment. Cell lysates were harvested 72 h later, and IR-induced apoptosis was detected by immunoblotting using an antibody against cleaved PARP.

For determine whether FATS expression was functionally associated with response to RT, we manipulated FATS expression using siRNA in breast cancer cell lines and assessed sensitivity to RT *in vitro*. First, MCF7 cells were transiently transfected with siRNAs targeting FATS or with a non-targeting control, and FATS expression was examined using Western blotting (Figure 4A). Expression of FATS was dramatically reduced by the targeted siRNAs. Next, we performed colony-forming assays with breast cancer cell lines representative of both MCF7 and MDA-MB-231 subtypes after transfecting a FATS-expressing vector [7], siRNAs targeting FATS and an empty vector as control, respectively, and after different doses of radiation from 0 to 8 Gy (Figure 4B). Cells surviving irradiation and maintaining sustained proliferative potential were quantified by counting individual colonies. FATS positive group significantly sensitized than Knock-down of FATS group in both cell lines.

In order to confirm the relationship between FATS mRNA levels and radiation sensitivity, we selected breast cancer cell line MDA-MB-231 and treated the cells with 6Gy ionizing radiation (IR) after transfecting a FATS-expressing vector [7], siRNAs targeting FATS and an empty vector as control, respectively. Radiation-induced apoptotic cells were examined by flow cytometry. Our data showed that the response to radiation was more sensitive after raising the mRNA levels of FATS in breast cancer cells (Figure 4C). Consistently, cleaved PARP, an apoptosis marker, was more pronounced after radiation treatment in FATS-positive cancer cells than that in Knock-down of FATS cancer cells (Figure 4C).

## DISCUSSION

There are currently no factors in use predictive for radiotherapy in breast cancer. Our study identified a potential marker, FATS, whose positive status prompted favorable breast cancer outcome. And this effect became more pronounced when patients had received adjuvant radiotherapy. Furthermore, our study showed that the prognostic effect of FATS mRNA level on disease-free survival was independent of the clinical factors, such as pathological stage, lymph node and ER. In addition, we found that forced expression of FATS sensitized radiation-induced apoptosis in breast cancer cells, further validating our clinical finding that FATS status is a biomarker in breast cancer to identify individuals likely to benefit from radiotherapy.

The linkage of insufficient FATS expression with radiation-induced tumorigenesis has been validated in mouse tumor samples through application of high-throughput DNA microarray technology and subsequently biological study on its function [7]. It's very interesting to find that FATS mRNA expression is a valuable predictive marker for clinical radiotherapy sensitivity. Although the application of high-throughput technologies to the analysis of clinical cancer specimens holds great promise in producing substantial advances in our understanding of the onset and progression of human breast cancer, results from currently available studies will be of limited help in guiding treatment decisions, since a plethora of repetitive sequences in human genome can raise the signal/background ratio in technology and the heterogenicity of patients in genetics and therapeutic treatments. Given that mouse genome is smaller and less complicate than human genome, our study emphasizes the advantage of dissecting the genetic aberrations in mouse tumor genome to identify new cancer biomarkers.

Strengths of this study include the identification of a tumor marker that may help to improve cancer therapies and attenuate the concern of SMN. Sarcoma risk increases significantly at radiation doses higher than 30 Gy, and the majority of epithelail cancers including breast cancer are only moderately radiosensitive and require a significantly higher dose of radiation (60–70 Gy) to achieve a radical cure [15–17]. Some types of cancer, e.g. renal cell cancer and melanoma, are notably radioresistant, that is, much higher doses are required to produce a radical cure than may be safe in clinical practice. Therefore, a notable decrease in SMN incidence might be expected with lower radiation doses. It will be important to evaluate whether the value of FATS expression in predicting the sensitivity to radiotherapy is applicable to other cancer types besides breast cancer. Distinguishing FATS status may thus be beneficial to improve radiotherapeutic protocols and reduce the risk of SMN.

Limitations of the present study include uncertainty about the effect of chemotherapy at the time of radiation for breast cancer. Although none of clinical samples derived from patients receiving neoadjuvant chemotherapy or taking radiosensitizing drugs such as cisplatin [18, 19], all patients received cyclophosphamide /fluorouracil-based chemotherapy after surgical resection, which compromised our ability to describe the prognostic value of FATS in predicting radiotherapy sensitization without the possible influence of chemotherapy. In addition, all cancer cases in our study had a very reduced FATs mRNA compared with normal breast tissue, the purpose of this paired design was to prevent the intervention of potential confounding factors to our results, and the relationship of FATs mRNA status between breast cancers and benign breast tumors needs to be proved. At the mention of the expression of FATs mRNA, there was no evidence indicated the threshold level of FATS mRNA that was required for FATS to be active in the cell lines, the method assessing the level of FATS mRNA based on ROC analysis was justified given some recent research [9–11], Besides, form the Table 1, we can know that negative status of FTAS was correlated with higher nuclear grade compared with positive FATS expression, which future prove the biological significance of this division using ROC curve. In the subgroup patients without radiation treatment, which were mostly node negative, the prognostic value of FATS status was not significant (Figure 3B). Although more patient samples need to be investigated, this suggests that FATS, a crucial inducer of p21 [8], might have a protective effect on nodal involvement during tumor development, which is confirmed by earlier reports that show a correlation between p21 expression and negative node status [20, 21] and that p21 expression alone fails to be of prognostic value [22]. Therefore, the prognostic value of FATS status in entire cohort (Figure 2 and Table 2) attribute specifically to its effect of radiotherapy sensitization in breast cancer (Figure 3 and Table 2). Whether FATS status has an independent prognostic value for patients managed by breast-conserving surgery and prospective breast irradiation remains to be investigated.

In conclusion, the quantitative detection of FATS mRNA level is helpful when making decision about the radiation treatment of breast cancer.

## MATERIALS AND METHODS

### Cell culture

Breast cancer cell line MDA-MB-231 was obtained from American Type Culture Collection (ATCC). Cells were grown in Dulbecco's modified Eagle's medium (Invitrogen) supplemented with 10% fetal calf serum.

### Clinical samples and tumor tissue bank

All breast tissues, including 156 primary tumors and 42 paired normal tissues, were collected from 156 breast cancer patients undergoing complete dissection of breast and axillary lymph nodes followed by adjuvant therapy at Tianjin Medical University Cancer Institute and Hospital between 2003 and 2004. All patients were received cyclophosphamide/fluorouracil-based adjuvant chemotherapy. Among them, eighty-one patients with breast cancer, who with ≥ 4 positive axillary lymph nodes or with T3 tumors with positive axillary lymph nodes, or with operable stage III tumors, were subjected radiotherapy according to clinical guidelines [23, 24]. Informed consent for sample collection was obtained according to protocols approved by the review boards of Institute and Hospital. Tissue samples were snap-frozen in liquid nitrogen and stored at −80°C in the Joint Tumor Tissue Bank of Tianjin Medical University Cancer Institute and Hospital (TMUCIH, China) and National Foundation for Cancer Research (NFCR, USA).

### Data collection

Tianjin Medical University Cancer Institute and Hospital review board approval was obtained for this retrospective study. The following data were abstracted from medical records: clinical stage, pathologic tumor size, lymph node status, nuclear grade, estrogen receptor (ER) status, progesterone receptor (PR) status, Her2 status, and adjuvant radiotherapy. ER and PR expression were determined by immunohistochemical staining (positive when more than 15% of the nuclei showed staining). Her2 status was defined as positive when more than 10% of the membrane showed staining in immunohistochemical assay. All samples were examined by hematoxylin–eosin staining on formalin-fixed paraffin-embedded sections, and only samples with 75% or more epithelial cells were selected for further study. Patients without documented death and with last known address were sent a follow-up questionnaire. All cases were followed up for more than years (from 60 to 88 months, median 72 months).

### RNA extraction and cDNA preparation

RNA was extracted with Trizol reagent (Invitrogen, Gaithersburg, MD, USA) according to manufacturer's instructions. The quality of RNA was assessed using formaldehyde agarose gel electrophoresis and quantified spectrophotometrically. Total RNA (5 μg) was used to perform reverse transcription for first-strand cDNA synthesis. In brief, RNA was denatured for 5 min at 65°C and cooled on ice in the presence of 0.5 μg Oligo(dT) (Invitrogen) and 10 mmol dNTPs (Invitrogen), followed by incubation with First-Strand Buffer (Invitrogen), 0.2 μmol DTT (Invitrogen), 40 U RNase inhibitor (Invitrogen) and 200U reverse transcriptase SuperScript II (Invitrogen) in total volume of 20 μl at 42°C for 60 min. Reactions were stopped by incubation at 70°C for 15 min.

### qRT-PCR

qRT-PCR analysis was performed using the Platium Quantitative PCR SuperMix-UDG system (Invitrogen) according to manufacturer's instructions. The expression of a housekeeping gene, glyceraldehyde-3-phosphate dehydrogenase (GAPDH), was quantified as control. The primers and Taqman probes of FATS (GenBank accession number, NM_001004298) were 5′-CATTCACATTCCTGGCTGGAGTTA-3′, 5′-CCTCTTGCTGCTTCCAGAAAATACT-3′, and 5′ (FAM)- CAGGGCAGTACACACAAA-(TAMRA)-3′. The primers and Taqman probes of GAPDH were 5′-GAAGG TGAAGGTCGGAGTC-3′, 5′-GAAGATGGTGATGG GATTTC-3′, and 5′ (FAM)-CAAGCTTCCCGTTCTCA

GCC-(TAMRA)-3′. Assays were carried out using the ABI 7500 TaqMan system (Applied Biosystems, Foster City, CA, USA). PCR experiment was carried out after incubation at 50°C for 2 min and denaturing at 95°C for 3 min, followed by 50 cycles of denaturing at 95°C for 30 s and annealing plus extension at 65°C for 1 min. Quantification of FATS gene expression in samples was determined by measuring cycle numbers at which the amounts of transcripts reached a fixed threshold (C_T_). The average C_T_ value of FATS gene was subtracted by that of GAPDH to obtain ΔC_T_, and the levels of FATS expression was calculated as 2^−ΔCT^.

### Western blot

Cells were transfected with a FATS-expressing vector [25] or an empty vector as control. After transfection for 24 h, cells were subjected to ionizing radiation (IR). Protein lysate was prepared 48 h later and subjected to Western blot analysis as described previously [8], using a primary antibody against cleaved PARP (Abcam).

### Flow cytometry

Flow cytometry analysis was performed as previously described [25].

### Statistical analysis

Paired Wilcoxon Rank Sum test was used to analyze the differences of mRNA expression between primary breast cancer and paired normal breast tissues. The association of FATS with various clinicopathologic factors was analyzed by χ2 test or Fisher's exact test. The cut-off value for distinguishing positive or negative expression of FATS mRNA in breast tissues was determined by receiver operating characteristic (ROC) curve and the area under the curve. Actuarial curves showing the probability of disease-free survival (DFS) and overall survival (OS) were defined using the Kaplan-Meier method [26] and *P* values were calculated using a log-rank test. Univariate analyses were carried out using the Pearson Chi-Square test. Multivariate analyses were carried out to evaluate the association of FATS expression with a priori defined potential predictors with *P* £ .05 in univariate analysis. Cox proportional hazards regression was used to examine whether FATS expression was an independent prognostic factor for survival when adjusting for other covariates (pathologic stage, lymph node, ER and PR) in multivariate analysis. All calculations were carried out using SPSS for Windows statistical software package (SPSS, Chicago, IL, USA).

### Colony-forming assays

These were performed essentially as described previously. Cell lines were transfected in T25 cm^2^ tissue culture flasks and cultured for 48 h as normal. Irradiation was then performed using a 320 System Irradiator (320 kV x-ray source; NDT Equipment Suppliers, UK). Cells were irradiated with single fraction of 0, 2, 4, 6, and 8 Gy and cultured as normal for a further 4 h. Each flask of cells was then seeded into triplicate 10 cm^2^ tissue culture plates. Cells were seeded at different densities according to cell type and radiation exposure in order to achieve an assessable number of colonies. The cells were then cultured undisturbed for 14 days. Cells were fixed and stained in 5 mg/ml Crystal violet, 50% Methanol, 20% Ethanol (20°C, 20 s) before being rinsed in water twice. Calculation of survival fractions (SF) was performed using the equation: SF = colonies counted/cells plated × (PE/100), where PE is a measure of individual plating efficiency.
